# Exploring Alternative Radiolabeling Strategies for Sialic Acid-Binding Immunoglobulin-Like Lectin 9 Peptide: [^68^Ga]Ga- and [^18^F]AlF-NOTA-Siglec-9

**DOI:** 10.3390/molecules23020305

**Published:** 2018-01-31

**Authors:** Olli Moisio, Riikka Siitonen, Heidi Liljenbäck, Elli Suomela, Sirpa Jalkanen, Xiang-Guo Li, Anne Roivainen

**Affiliations:** 1 Turku PET Centre, University of Turku, FI-20521 Turku, Finland; oamois@utu.fi (O.L.); ralsii@utu.fi (R.S.); halilj@utu.fi (H.L.); elli.a.suomela@utu.fi (E.S.); xiali@utu.fi (X.-G.L.); 2 Turku Center for Disease Modeling, University of Turku, FI-20520 Turku, Finland; 3 MediCity Research Laboratory, University of Turku, FI-20520 Turku, Finland; sirjal@utu.fi; 4 Turku PET Centre, Åbo Akademi University, FI-20521 Turku, Finland; 5 Turku PET Centre, Turku University Hospital, FI-20521 Turku, Finland

**Keywords:** aluminum fluoride, fluorine-18, gallium-68, inflammation, positron emission tomography, Siglec-9, VAP-1

## Abstract

Amino acid residues 283–297 from sialic acid-binding immunoglobulin-like lectin 9 (Siglec-9) form a cyclic peptide ligand targeting vascular adhesion protein-1 (VAP-1). VAP-1 is associated with the transfer of leukocytes from blood to tissues upon inflammation. Therefore, analogs of Siglec-9 peptide are good candidates for visualizing inflammation non-invasively using positron emission tomography (PET). Gallium-68-labeled 1,4,7,10-tetraazacyclododecane-*N,N′,N″,N‴*-tetraacetic acid (DOTA)-conjugated Siglec-9 has been evaluated extensively for this purpose. Here, we explored two alternative strategies for radiolabeling Siglec-9 peptide using a 1,4,7-triazacyclononane-triacetic acid (NOTA)-chelator to bind [^68^Ga]Ga or [^18^F]AlF. The radioligands were evaluated by in vivo PET imaging and ex vivo γ-counting of turpentine-induced sterile skin/muscle inflammation in Sprague-Dawley rats. Both tracers showed clear accumulation in the inflamed tissues. The whole-body biodistribution patterns of the tracers were similar.

## 1. Introduction

Inflammation is associated with several diseases including atherosclerosis, rheumatoid arthritis, and certain cancers. Positron emission tomography (PET) imaging offers a valuable diagnostic and research tool for in vivo detection and quantification of inflammation in a non-invasive manner [1,2]. The most commonly used PET-radiotracer, 2-deoxy-2-[^18^F]-fluoro-d-glucose ([^18^F]FDG), has been employed for many indications involving inflammation [3], including the assessment of atherosclerotic plaques [4]. Although glucose uptake in regions of inflammation is higher than normal, [^18^F]FDG is not a specific tracer for inflammation, which can lead to false positives. Thus, development of alternative, inflammation-specific PET radiotracers is justified. Potential alternative targets for PET imaging of inflammatory conditions include somatostatin receptors [5,6,7], 18 kDa translocator protein [8], B-lymphocyte antigen CD20 [9], and, more recently, CXCR4 chemokine receptors [10,11,12].

We are pursuing vascular adhesion protein 1 (VAP-1, also known as primary amine oxidase [AOC3, EC 1.4.3.21]) as a target for PET imaging of various indications involving inflammation. VAP-1 is involved in the molecular adhesion cascade, which leads to the transfer of circulatory leukocytes into tissues undergoing inflammatory responses. More precisely, the monoamine oxidase activity of VAP-1 enables the adhesion, rolling, and transmigration of leukocytes during the cascade [13,14]. An especially beneficial property of VAP-1 in terms of PET imaging is that, under normal physiological conditions, VAP-1 is stored in intracellular storage granules and is transported upon inflammation to the endothelial cell surface, readily accessible to circulatory PET-ligands. Sialic acid-binding immunoglobulin-like lectin 9 (Siglec-9) has been identified as a leukocyte ligand binding to the enzymatic groove of VAP-1 [15].

A small cyclic peptide consisting of Siglec-9 amino acid residues 283–297 (amino acid sequence “CARLSLSWRGLTLCPS”) is currently the most thoroughly investigated PET-ligand for detection of VAP-1. The radiotracers containing this peptide sequence have been abbreviated as Siglec-9 for simplicity, and further mentions of Siglec-9 in this article refer to the disulfide-bridged “CARLSLSWRGLTLCPSK”-sequence. Thus far, 1,4,7,10-tetraazacyclododecane-*N,N′,N″,N‴*-tetraacetic acid (DOTA)-conjugated gallium-68 labeled peptide, [^68^Ga]Ga-DOTA-Siglec-9, has been successfully prepared [16] and employed in various preclinical inflammatory disease models such as synovitis [17], atherosclerosis [18], peri-implant tissue responses and staphylococcal infections [19], and acute respiratory distress syndrome [20].

Although [^18^F]FDG lacks specificity towards inflammation, ^18^F itself has superior properties compared to ^68^Ga when PET image quality is concerned. The radionuclide ^18^F offers a shorter positron range and a higher positron yield than ^68^Ga, resulting in greater spatial resolution [21]. The less optimal properties of ^68^Ga can limit spatial resolution, especially considering high-resolution PET imaging of small animals such as rats or mice. Siglec-9 has previously been labeled with 5-deoxy-5-[^18^F]fluoro-d-ribose ([^18^F]FDR) as a prosthetic agent, resulting in the radiotracer [^18^F]FDR-Siglec-9 [22,23]. We propose that the labeling process of Siglec-9 with ^18^F can be further simplified by using aluminum-fluoride-18 ([^18^F]AlF) for radiofluorination. Similar to [^68^Ga]Ga^3+^, the [^18^F](AlF)^2+^ complex can be captured by a suitable chelator such as triazacyclononane-1,4,7-triacetic acid (NOTA) [24].

**Figure 1:**
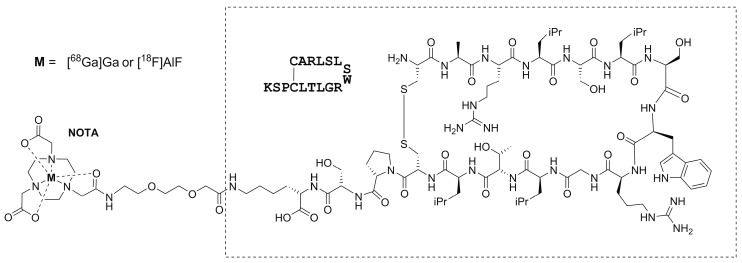
The molecular structure of NOTA-Siglec-9. The molecular weights of [^68^Ga]Ga- and [^18^F]AlF-NOTA-Siglec-9 are 2387.66 and 2364.71, respectively.

Here, we present two alternative strategies for the radiolabeling of Siglec-9 using NOTA-conjugated Siglec-9 as the precursor (Figure 1). Both [^68^Ga]Ga- and [^18^F]AlF-NOTA-Siglec-9 were synthesized using the described procedures and evaluated by PET imaging and ex vivo tissue analysis of turpentine oil-induced acute sterile skin/muscle inflammation in male Sprague-Dawley rats.

## 2. Results and Discussion

### 2.1. Radiosynthesis

The radiosyntheses of both [^68^Ga]Ga- and [^18^F]AlF-NOTA-Siglec-9 were very straightforward and provided good radiochemical yields and purities. Average activity yields of 53 ± 2% for [^68^Ga]Ga-NOTA-Siglec-9 and 26 ± 3% for [^18^F]AlF-NOTA-Siglec-9 were achieved with radiochemical purities of >98% for both tracers. Both distribution coefficient (Log *D*) and in vivo plasma protein binding after 60 min were very similar between the two PET radiotracers (Table 1). For both radiotracers, NAP^TM^ size exclusion columns were used for purification.

### 2.2. PET Imaging

**Figure 2:**
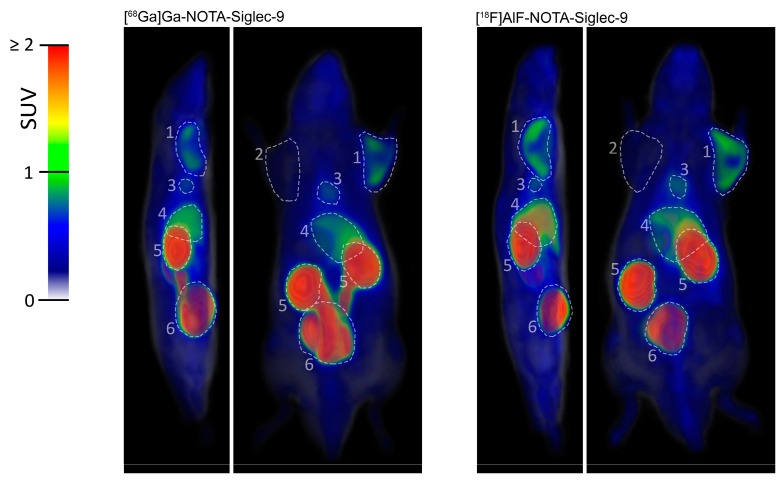
Sagittal (**left panels**) and coronal (**right panels**) maximum intensity projection PET images of [^68^Ga]Ga- and [^18^F]AlF-NOTA-Siglec-9 biodistribution in rats. The images are averaged from time frames 10 to 60 min and normalized to the same standardized uptake value (SUV) scale. Highlighted regions of interest: (1) inflamed area; (2) control area; (3) heart; (4) liver; (5) kidneys; and (6) urinary bladder.

**Figure 3:**
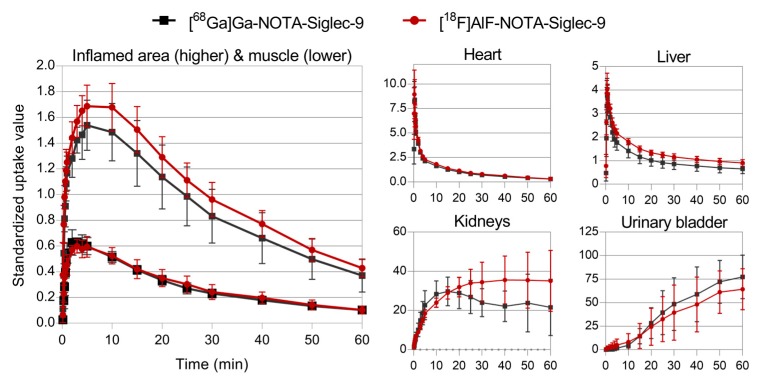
Time-activity curves for [^68^Ga]Ga-NOTA-Siglec-9 and [^18^F]AlF-NOTA-Siglec-9 (*n* = 4).

**Figure 4:**
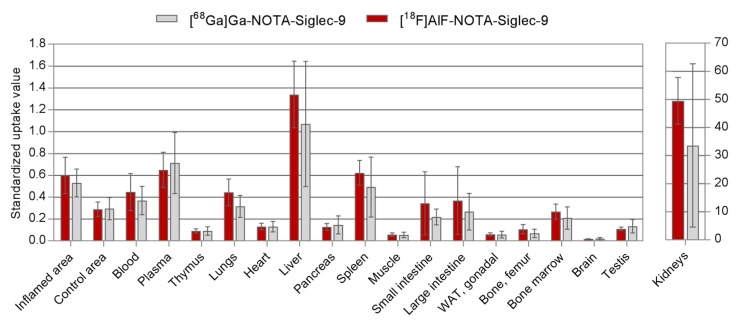
Standardized uptake values acquired 60 min post-injection by ex vivo γ-counting of excised tissues (*n* = 7).

The dynamic PET imaging showed clear accumulation of both tracers in the inflamed tissues. Although the radioactivity concentration of inflamed tissue seemed slightly higher for [^18^F]AlF-NOTA-Siglec-9, the image quality of the ^68^Ga- and ^18^F-labeled tracers was comparable (Figure 2). With both tracers, the excess radioactivity was excreted through the kidneys to the urinary bladder with moderately high uptake in the liver. This is characteristic of Siglec-9-based ligands. Bone uptake was low, indicating minimal in vivo defluorination or release of ^68^Ga. While other degradation products cannot be ruled out, the amount of free radioisotope ions may be asumed to be low for both radiotracers. Selected volumes of interest (VOI) were isolated from the 3D dynamic PET data to produce time-activity curves (TACs, Figure 3) for inflamed tissue, muscle, heart, liver, urinary bladder, and kidneys. The standardized uptake values (SUVs) acquired from the TACs were in line with those acquired from ex vivo analysis of dissected tissues (Figure 4).

### 2.3. Ex Vivo Radioactivity Distribution

**Figure 5:**
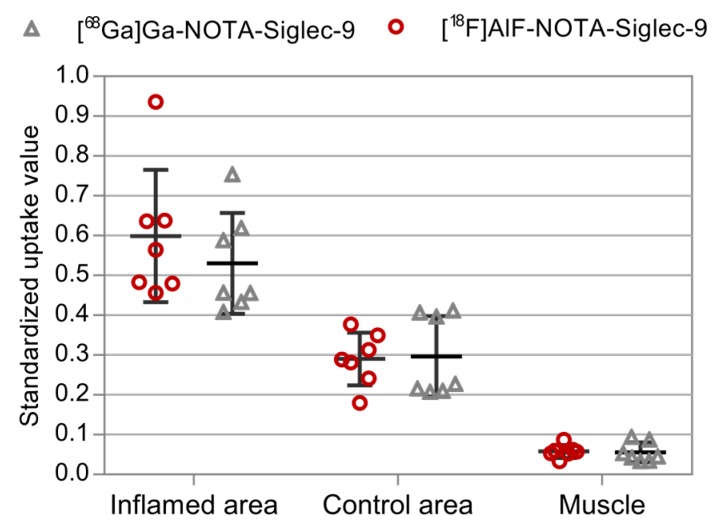
Inflamed area (skin/muscle from the right foreleg, control area (skin from the left foreleg), and healthy muscle (hind leg) SUVs (ex vivo, 60 min after injection, *n* = 7).

SUVs acquired by ex vivo γ-counting of excised tissue samples (Figure 4 and Figure 5) verified the findings from the PET imaging. Both tracers showed characteristics typical of previous Siglec-9 tracers, with low bone uptake. Radioactivity concentration in the inflamed area was elevated, with an inflamed tissue-to-control area ratio of 1.8 ± 0.3 (*n* = 7) for [^68^Ga]Ga-NOTA-Siglec-9 and 2.1 ± 0.3 (*n* = 7) for [^18^F]AlF-NOTA-Siglec-9. Inflamed tissue-to-muscle ratios were 10.2 ± 2.4 (*n* = 7) and 10.4 ± 1.4 (*n* = 7), respectively.

## 3. Materials and Methods

Chemicals and NAP^TM^ columns (GE Healthcare) were acquired from Sigma-Aldrich (St. Louis, MO, USA). NOTA-Siglec-9 precursor was obtained from Peptide Specialty Laboratories (Heidelberg, Germany) as a custom synthesis. ^68^Ga was obtained from a ^68^Ge/^68^Ga generator (Eckert & Ziegler, Valencia, CA, USA) by elution with 0.1 M HCl. Traceselect water (Honeywell, Morristown, NJ, USA) was used for radiosynthesis. [^18^F]-Fluoride was produced with a cyclotron. A High-Resolution Research Tomograph (HRRT, Siemens, Knoxville, TN, USA) was used for PET imaging. A Wizard γ-counter (1480 Wizard 3”, PerkinElmer/Wallac, Turku, Finland) was used for radioactivity counting for ex vivo tissue samples.

### 3.1. [^68^Ga]Ga-NOTA-Siglec-9 Radiosynthesis

^68^GaCl_3_ was collected from the fraction with the highest radioactivity, and 1 mL was added to two low-bind microcentrifuge tubes (Eppendorf, Hamburg, Germany), both containing sodium acetate (18 mg). The pH of the reaction mixture was adjusted to 3–4 by addition of 47 µL 2 M HCl to each vial. NOTA-Siglec-9 peptide (10 nmol, 10 µL of 1 mM solution in water) was added to both vials. The reaction mixture was incubated for 15 min at 60 °C. The mixture was then cooled and loaded onto a NAP-25 size exclusion column and eluted with phosphate-buffered saline (PBS). Radiochemical purity was analyzed with high-performance liquid chromatography (HPLC). The HPLC conditions were as follows: 150 × 4.60 mm Jupiter 5 µ C18 300 Å column (Phenomenex, Torrance, CA, USA); flow rate  = 1 mL/min; wavelength λ  =  220 nm; solvent A  =  0.1% trifluoroacetic acid (TFA) in water; solvent B  =  0.1% TFA in acetonitrile; gradient: during 0–2 min 82% A and 18% B; during 2–11 min from 82% A and 18% B to 40% A and 60% B; during 11–15 min from 40% A and 60% B to 82% A and 18% B; during 15–20 min 82% A and 18% B. The radio-HPLC system consisted of LaChrom Instruments (Hitachi; Merck, Darmstadt, Germany) and of a Radiomatic 150TR flow-through radioisotope detector (Packard, Meriden, CT, USA).

### 3.2. [^18^F]AlF-NOTA-Siglec-9 Radiosynthesis

[^18^F]-Fluoride (42 µL in physiological saline) was added to a reaction vial preloaded with CH_3_CN (76 µL) and AlCl_3_ (21 µL, 2 mM in 1.0 M CH_3_CO_2_Na buffer, pH 4.0). The reaction mixture was incubated at room temperature for 3 min. Polypropylene glycol (18 µL) and precursor NOTA-Siglec-9 (26 µL in water, 52.5 nmol) were then added, and the mixture was incubated at 100 °C for 15 min. After incubation, the mixture was cooled to 35 °C. The mixture was pushed through an Oasis 30 mg HLB cartridge (Waters, Milford, MA, USA), and the cartridge was washed with 7 mL water. The product was eluted from the cartridge with 0.05% formic acid in ethanol (0.5 mL) and subsequently loaded onto a NAP-5 column. The column was eluted with PBS, and the product was collected. Radiochemical purity was determined as described in Section 3.1.

### 3.3. Distribution Coefficient (Log D)

The distribution coefficient (Log *D*) was measured by adding 5 kBq of tracer into a mixture of PBS (pH 7.6) and 1-octanol (1:1, *v/v*). The solution was mixed thoroughly for 5 min at room temperature. After separation of phases by centrifugation (12,000× *g*, 6 min), 100 µL aliquots of each phase layer were taken for γ-counting to determine Log *D*. The test was repeated three times.

Log *D* was calculated as $\mathrm{Log}\;D=\log10\;\frac{counts\;per\;minute\;in\;octanol}{counts\;per\;minute\;in\;PBS}$.

### 3.4. PET Imaging of Sterile Skin/Muscle Inflammation

Male Sprague-Dawley rats were used in the experiments with approval from the National Animal Experiment Board in Finland and the Regional State Administrative Agency for Southern Finland and in accordance with the European Union directive relating to the conduct of animal experimentation. A total of 15 rats with sterile skin/muscle inflammation (weight 360 ± 61 g) were used for the studies. Eight rats were imaged and analyzed ex vivo, and seven additional rats were subjected to ex vivo analysis only.

The rats were subcutaneously injected with 50 µL turpentine oil (Sigma-Aldrich) into the right shoulder area (foreleg) to induce skin/muscle inflammation, which was allowed to develop for 24 h prior to PET imaging. For imaging and ex vivo analysis, the rats were anesthetized using isoflurane and intravenously injected with 17.7 ± 3.4 MBq of one of the two radiotracers via the tail vein cannula. Immediately after injection a dynamic 60-min PET scan was performed on four rats for [^68^Ga]Ga-NOTA-Siglec-9 and four rats for [^18^F]AlF-NOTA-Siglec-9. The data were acquired in list-mode and iteratively reconstructed with a 3-D ordered subsets expectation maximization algorithm with 8 iterations, 16 subsets, and a 2-mm full-width at half-maximum post-filter into 6 × 10 s, 4 × 60 s, 5 × 300 s, and 3 × 600 s time frames. After imaging, the rats were euthanized, and various tissues, including inflamed skin/muscle (inflamed area) from the right foreleg, skin from the left foreleg (control area), and healthy muscle from the left hind leg, were excised, weighed, and measured with a γ-counter. The ex vivo radioactivity measurements were decay-corrected from the time of injection, and the results were expressed as SUV.

For quantification of PET image data, VOIs were defined on inflamed area, control area, heart, kidneys, liver and urinary bladder using Carimas software version 2.9 (Turku PET Centre). Results were expressed as SUVs and TACs. SUVs were calculated as the average radioactivity concentration of the VOIs corrected for the injected radioactivity dose and animal body weight.

### 3.5. Plasma Protein Binding Measurements

Plasma protein binding was measured from plasma samples taken from 60 min post-injections. Plasma proteins were precipitated by addition of acetonitrile (1:1, *v/v*) to the plasma samples, followed by centrifugation at 14,000× *g* for 5 min at room temperature and separation of supernatant. Radioactivity of both the precipitate and the supernatant was measured by γ-counting.

## 4. Conclusions

In conclusion, [^68^Ga]Ga- and [^18^F]AlF-NOTA-Siglec-9 were produced with high radiochemical purity and molar activity to achieve successful preclinical in vivo PET imaging of sterile inflammation. The foci of the inflamed tissues were clearly visualized. The results observed with in vivo PET imaging were further confirmed by γ-counting of ex vivo tissues and biodistribution proved similar to previously studied Siglec-9 PET-ligands. Importantly, low bone uptake was indicative of minimal in vivo defluorination, which can be a concern with some ^18^F-labeled ligands. Therefore, the two radiolabeling methods described here are good alternatives for producing Siglec-9 peptide-based PET tracers for imaging of inflammation.

## Figures and Tables

**Table 1:** Properties of [^68^Ga]Ga-NOTA-Siglec-9 and [^18^F]AlF-NOTA-Siglec-9.

	[^68^Ga]Ga-NOTA-Siglec-9	[^18^F]AlF-NOTA-Siglec-9
Yield (%, *n* = 3)	53 ± 2 AY ^1^; 64 ± 1 RCY ^2^	26 ± 3 AY ^1^; 39 ± 1 RCY ^2^
Radiochemical purity (%, *n* = 3)	98.5 ± 0.2	98.2 ± 1.4
Total synthesis time (minutes)	30	60
Molar activity (MBq/nmol, *n* = 3)	5.0 ± 0.2	8.8 ± 0.9
Log *D* (*n* = 3)	−3.2 ± 0.3	−2.3 ± 0.1
Plasma protein binding (%, *n* = 5) ^3^	31.4 ± 3.4	37.4 ± 0.9

Results are expressed as mean ± SD. ^1^ AY = activity yield (non-decay corrected); ^2^ RCY = radiochemical yield (decay-corrected); ^3^ Radioactivity of plasma proteins divided by total plasma radioactivity from blood samples collected 60 min after intravenous injection in rats.
